# Epidemiology and Molecular Profile of Mucosal Melanoma: A Population-Based Study in Southern Europe

**DOI:** 10.3390/cancers14030780

**Published:** 2022-02-03

**Authors:** Anna Carbó-Bagué, Jordi Rubió-Casadevall, Montserrat Puigdemont, Arantza Sanvisens, Glòria Oliveras, Mònica Coll, Bernat del Olmo, Ferran Perez-Bueno, Rafael Marcos-Gragera

**Affiliations:** 1 Medical Oncology Department, Catalan Institute of Oncology, Hospital Universitari Josep Trueta, Av. França S/N, 17007 Girona, Spain; jrubio@iconcologia.net; 2 Research Group on Descriptive Epidemiology, Genetics and Cancer Prevention, Girona Biomedical Research Institute (IDIBGI), 17190 Girona, Spain; mpuigdemont@iconcologia.net (M.P.); asanvisens@carrerasresearch.org (A.S.); rmarcos@iconcologia.net (R.M.-G.); 3 School of Medicine, University of Girona (UdG), 17003 Girona, Spain; bdelolmo@gencardio.com; 4 Epidemiology Unit and Girona Cancer Registry, Oncology Coordination Plan Department of Health Government of Catalonia, Catalan Institute of Oncology, 17004 Girona, Spain; 5 Pathology Department, Hospital Josep Trueta, Catalan Institute of Health, 17007 Girona, Spain; goliveras@iconcologia.net (G.O.); fperezbueno.girona.ics@gencat.cat (F.P.-B.); 6 Cardiovascular Genetics Center, Biomedical Research Institute of Girona (IDIBGI), 17190 Salt, Spain; mcoll@gencardio.com

**Keywords:** mucosal melanoma, epidemiology, incidence, survival, DNA mutational analysis, NRAS

## Abstract

**Simple Summary:**

There are few population-based studies focused on the epidemiology of mucosal melanoma, a rare neoplasm. Its poor prognosis, the different etiology from cutaneous melanoma and the lack of effective treatment beyond corrective surgery, make the knowledge of the mutational profile of this type of cancer a useful tool in understanding its natural history and also for the investigation of new target therapies. The aim of our population-based study is to analyze the incidence and survival of mucosal melanoma, which mainly arises from the head and neck sphere, genitourinary tract and rectal area, and to carry out the mutational analysis of selected cases. We used the Girona Cancer Registry database, which registered all cancer cases in Girona, a province of Spain in southern Europe, during the period of 1994–2018.

**Abstract:**

Background: Mucosal melanoma is a rare neoplasm on which few epidemiological population-based studies have been published. A good surgical approach is the standard treatment, but the prognosis is worse than that of skin melanoma. The analysis of mucosal melanoma’s mutational profile can help to develop target therapies in advanced disease or adjuvant settings. Methods: We analyzed the database of the Cancer Registry of Girona, a region located in the north-east of Spain, in the period of 1994–2018. We selected cases of primary invasive melanoma, excluding those located in the skin, eye, central nervous system and an unknown primary site. Epidemiological analysis included incidence and survival. Mutational profile analysis was performed with a custom gene panel. Results: Forty-two patients were identified: 14 (33%) had vulvar-vaginal melanoma, 15 (35.7%) had rectal melanoma, 12 (28.6%) had melanoma located in the head and neck sphere and 1 male patient had a urethral melanoma. European age-standardized incidence rates for vulvar-vaginal, rectal and head and neck melanoma were 0.09, 0.1 and 0.09 cases/100,000 inhabitant-years, respectively. Five-year observed survival rates were 37.5%, 20% and 25% for these types of cancers. NRAS Q61 was the most frequent mutation found. Conclusion: Our study confirms the steady incidence and low survival of mucosal melanomas in a region of southern Europe. NRAS and NF1 play a role in the molecular landscape of mucosal melanoma. MEK and PI3K/mTOR inhibitors could be reasonable treatment options and are being studied in clinical trials.

## 1. Introduction

Melanomas of the mucosa (MMs) are neoplasms that arise from melanocytes of the epithelium of the otorhinolaryngological sphere (oral and nasal cavities), conjunctiva, genitourinary tract (especially in the vulvovaginal area) and anorectal area. 

MMs are a very rare type of cancer, with a much lower incidence and worse prognosis compared to cutaneous melanomas (CMs) and account for approximately 1.2% of all melanomas. Five-year survival of MM patients is less than 25%, and 23% of patients are diagnosed with metastasis [1]. MM patients have a median survival of 9 months and the worst prognosis compared with other melanoma subgroups such as uveal, acral, non-acral cutaneous and unknown primary melanoma [2].

Clinical guidelines strongly recommend testing BRAF, NRAS and KIT in all melanomas. The BRAF mutation in MM has been reported in 3–15% of cases, while it has been reported in nearly 50% of CM cases. Mutations of KIT can be identified in 7–17% of cases, a much higher value than in cutaneous melanoma, especially in vulvovaginal melanomas, where they may be identified in 30% of cases [1]. Mutations in NRAS have been found in 15–20% of mucosal melanomas, such as cutaneous ones. 

Our aim is to conduct a population-based study of the incidence and survival of mucosal melanomas in Girona’s province, a region of southern Europe, from 1994 to 2018 and to perform a genetic analysis to determine the molecular landscape of these neoplasms. 

## 2. Materials and Methods

### 2.1. Study Cohort

This is a retrospective cohort population-based study. We analyzed the database from the Girona Cancer Registry (GCR), a population-based cancer registry in Girona’s province, located in the north-east of Spain, which started case registration in 1994. The population covered is 749,656 inhabitants according to the 2018 census. GCR cases are registered according to the International Agency of Research on Cancer (IARC) guidelines with a completeness of 95.0% (http://ico.gencat.cat/web/.content/minisite/ico/professionals/documents/registre_cancer_girona/arxius/CanGir-2013-17.pdf, accessed on 28 November 2021). The International Classification for Diseases-Oncology, Third Edition (ICD-O-3), was used to register cases [3].

We restricted our analysis to cases of primary invasive melanoma (ICD-O-3, histological codes: 8720-8723, 8730, 8740-8746 and 8761-8774) and excluded those located on the skin (code C44), in an unknown primary site (C80.9) and in the eye and central nervous system (C69-C72). Patients were eligible if diagnosed from the 1 January 1994, to 30 December 2018. 

We obtained paraffin-embedded tissue samples from all the hospitals in our province by previous contact and agreement with the collaborating pathology labs. Samples with the highest proportion of tumor cells were selected by pathologists from the University Hospital Josep Trueta. 

### 2.2. Descriptive Epidemiology

Descriptive statistics were expressed as median and interquartile ranges (IQR) for quantitative variables and as absolute frequencies and percentages for qualitative variables. Crude (CR) and age-standardized incidence rates using the 2013 European standard population (ASIR_e_) and world standard population (ASIRw) were calculated and expressed per 100,000 person-years. For the survival analysis, we calculated follow-up time from diagnosis to patients’ last vital status recorded. To obtain these data, we reviewed hospital clinical reports and/or the Mortality Registry of Catalonia and the Spanish National Death Index. Vital status was updated on the 31st of August 2021. The observed survival (OS) estimates were analyzed by Kaplan–Meier method using R software v.3.6.2. 

### 2.3. DNA Extraction, Library Preparation and Sequencing

DNA extraction was performed with cobas sample kits. We excluded almost half of the cases either for not having enough archived tissue or due to bad quality of DNA. DNA quality and quantity were assessed using a genomic DNA ScreenTape on a TapeStation 2100 instrument from Agilent Technologies. We found 24 out of 42 cases with proper DNA quality for NGS analysis (60% of the cohort): *n* = 9 rectal melanoma, *n* = 8 head and neck (5 nasal and 3 pharyngeal) melanoma, *n* = 7 vulvar-vaginal.

Old paraffin tissue samples have high degradation levels of DNA; therefore, a screen test that served as quality control for DNA or RNA samples was used. 

We used a custom gene panel, designed and validated internally at Gencardio Diagnostics (University of Girona-IDIBGI). This panel covers the entire exonic regions from the following 44 genes: AKT1, AKT3, ALK, APC, BRAF, BRCA1, BRCA2, CDKN2A, CTNNB1, DDR2, EGFR, ERBB2, ERBB3, ERBB4, FGFR1, FGFR2, FGFR3, NF1, HRAS, JAK1, JAK2, KIT, NRAS, MAP2K1, MAP2K2, MET, MYC, NRAS, NTRK1, NTRK2, NTRK3, PDGFRA, PIK3CA, POLD1, PLE, PTEN, RB1, RET, ROS1, SLC34A2, SOX2, STK11, TERT and TP53. In addition, the panel covers hotspots for 8 known fusion genes— ALK, BRAF, EGFR, FGFR1, FGFR2, FGFR2, RET and ROS1—and a selection of common SNVs to create a backbone for Copy Number Alteration (CNA) detection. 

Sample library preparation was performed following the Sureselect XT HS Target Enrichment System (Agilent Technologies). Upon enzymatic fragmentation and adapter and index ligation, DNA fragment size and concentration were assessed using a TapeStation instrument. DNA fragments were hybridized using biotinylated RNA probes (Agilent Technologies) corresponding to the regions of interest of the panel design. The capture was performed using streptavidin-coated beads, and the captured DNA was PCR-amplified. To improve the on-target capture, a second hybridization and capture were performed. Finally, the specific molarity of each library was checked in the TapeStation instrument in order to multiplex the samples. Libraries were sequenced on a MiSeq instrument using 2 × 76 base pairs read length (Illumina, San Diego, CA, USA).

### 2.4. NGS-Analysis

NGS analysis was performed using a custom bioinformatics pipeline available at https://github.com/GENCARDIO/GC_NGS_PIPELINE (accessed on 28 November 2021). Raw FASTQ files were preprocessed to remove low-quality bases and adapters using fastp (v0.21.0). Read alignment to the human reference genome (GRCh37/hg19) was performed using the Burrows-Wheeler Aligner (BWA-MEM; v0.7.17). Sequencing and optical duplicates were removed with Picard (v2.18.9). SNV detection was performed using Mutect2 (v4.2.2.0) in combination with Lancet (v1.1.0). INDELs (<50 bp) were detected using Lancet. Structural Variants (SVs) were detected using Manta (v1.6.0). Copy Number Alteration (CNA) detection was performed using CNVkit (v0.9.8). Variants displaying significant strand bias due to FFPE artifacts were removed using GATK FilterByOrientationBias tool. Only variants with a Variant Allele Frequency (VAF) higher than 10% were kept for downstream annotation. Variant annotation was performed using Variant Effect Predictor (Ensembl release 101), with the selection of MANE isoforms. General population frequencies were annotated with gnomAD v2.1.1, ExAC and 1000Genomes. Clinical annotation levels of evidence were extracted from CIViC. Gene fusions were annotated with chimerKB v4. 

All significant variants were manually checked with the Integrative Genomics Viewer (https://www.broadinstitute.org/software/igv/home, accessed on 28 November 2021).

## 3. Results

### 3.1. Descriptive Epidemiology

Forty-two patients with MM were identified in the cohort: 14 female patients with vulvar-vaginal melanoma, 15 rectal melanoma cases, 12 patients with head and neck melanoma (eight nasal and four pharyngeal) and 1 male patient with a penile urethral melanoma. 

The ASIRw of MM in Girona between 1994 and 2018 for both sexes was 0.14. It was 0.10 for males and 0.16 for females, which was higher because of the number of vulvar-vaginal melanomas according to other articles in the literature. 

Table 1 shows sex and site distribution with incidence rates of the whole cohort and of each subgroup of patients in CR, ASIRw and ASIRe, for men, women and both sexes.

Table 2 shows the OS results. We summarize the 5-year OS for all stages for the whole cohort and each subgroup. OS at 5 years was 7.7% in men, 34.5% in women and 26% for both sexes.

Women survived longer, and vulvar-vaginal melanoma was the subgroup with better survival. This subgroup was the one in which we observed a more standardized diagnostic and treatment approach, probably due to the feasibility of applying the known evidence extrapolated from the management of CM [4]. Table 3 summarizes the characteristics of the patients with vulvovaginal melanoma. Staging has been extrapolated from the cutaneous melanoma TNM, 7th Edition from the American Joint Committee on Cancer system since there is no specific staging for MM. 

### 3.2. Genetics

From the 42 cases of the cohort, 24 cases (60%) were suitable for NGS analysis: nine rectal melanomas, eight head and neck (five nasal and three pharyngeal) melanomas and seven in the vulvar-vaginal area. In Table 4, results from the genetic profiling are shown. All pathogenic somatic mutations found in the assay are represented. 

Pathogenic somatic mutations of the studied genes were identified in 18 cases (75%). Eight cases had only one mutation, three cases had two mutations, five cases had three mutations and two had four mutations. 

Only one patient had a BRAF G596R mutation, which is more typically seen in lung cancer and is present in 0.02% of all malignant solid tumors [5]. There are open clinical trials for this type of mutation, also in melanoma (www.mycancergenome.org Accessed on 28 November 2021).

NRAS mutations were found in three cases (12.5%), and one KRAS G12C mutation, for which there are clinically tested drugs, was found. NF1 mutations dominated, with seven cases (29%) predominantly in the rectum. KIT mutation L576P exon 11 was found in one case of vulvar-vaginal melanoma. TP53 mutations, widespread in solid tumors, are present in MM too: in the present cohort, four cases were found (16.6%).

Amplifications of MYC were the most frequent copy number variations (CNV) with an average number of five copies found in eight patients. 

Several other mutations were found that are very uncommon in melanomas and are of uncertain meaning, such as POLE mutations, which are well-characterized in other solid tumors. One patient with vulvar MM had a frameshift mutation in the BRCA1 gene with a variant allele frequency (VAF) of 80%. Another patient with rectal MM had a CDKN2A mutation with VAF of 45%. For this reason, an underlying germline mutation was suspected, and genetic counseling was recommended. 

## 4. Discussion

There are few population-based studies published in the literature focused on mucosal melanoma, meaning that its epidemiology remains sparsely analyzed. MMs are underreported, which makes it difficult to develop large studies. Treatment is also not well-standardized, although this is not the purpose of this study. 

Beaudoux et al. published the epidemiology of mucosal melanoma in the region of Champagne-Ardenne in France in the period of 2001–2014. They identified 39 cases of MM, including those arising in the eye. Their incidence of 0.18/100,000 inhabitants-year in ASIRw is similar to ours of 0.14/100,000 inhabitants-year. The five-year survival for all stages in the French study was 31.8%, slightly higher than that of our study, 26.2% [6]. 

However, Beaudoux et al.’s study has an important difference compared to ours: they included conjunctival melanoma. We excluded conjunctival melanoma (C69.0) from our analysis mainly for two reasons: first, it is subject to the bias of a non-specific registration, since it belongs to the same location in ICD-O-3 as uveal or choroidal melanoma (C69.3 and C69.4), which we cannot consider mucosal melanoma. In addition, a significant number of diagnoses used to complete the GCR database in this site are coded as C69.9 without further specification of sublocation. Second, conjunctival melanoma is genetically and biologically different, while head and neck, rectal and vulgo-vaginal melanomas are molecularly similar, and it is the only mucosal melanoma exposed to the sun, as all other mucosal melanomas are found in non-exposed sites. Therefore, there is no known modifiable risk factor. This could explain the higher frequency of BRAF-V600E mutations found in conjunctival melanoma published in other studies, closer to the cutaneous one [7]. 

Bishop et al. published results from the Surveillance Epidemiology and End Results Program (SEER) in the period of 1988–2010. They identified 2755 cases of MM and reported an incidence of 0.23/100,000 inhabitants-year and a 5-year survival for all stages of 34%. The authors also described better survival in vulvar melanomas than in other subgroups, with 40% of patients alive at 5 years [8].

Similar results to those of SEER were obtained in the California Cancer Registry, which analyzed 1824 mucosal melanomas diagnosed between 1994 and 2015 [9], and the North American Association of Central Cancer Registries, in a study where 1806 cases diagnosed in the period of 1996–2000 were analyzed [10].

Differences in the incidence between the population studies mentioned above and ours may be due to the inclusion of conjunctival melanomas or geographical variability, this study being the first population-based one in southern Europe, to our knowledge.

In our study, some information about clinical and pathological characteristics was missing in a considerable percentage of cases, not allowing us to perform a multivariate analysis for prognostic factors. The reason for the lack of information is mainly that the patients were often diagnosed in advanced disease and a surgical approach was not used. This limitation has also been observed in other studies, also with considerable missing data in pathological characteristics [5,10]. In contrast to primary CM, the value of pathological characteristics such as Breslow or ulceration as prognostic parameters has not been consistently attested in MM [5,10]. The prognostic or predicting factors of MM that cause unfavorable outcomes are not certain, although LDH level and performance status were found to be significant in a recently published survival meta-analysis [11,12].

We were able to describe characteristics of vulvar-vaginal melanoma cases, where the treatment approach is similar to CM. Our results are not far from those published by Aliteri et al. [9] in terms of survival but are lower than those obtained by Sanchez et al. [13] and Wolhmut et al. [14], who reported a 5-year OS of up to 50% for vulvar-vaginal melanoma. Indeed, vaginal melanoma has worse prognosis than vulvar melanomas [15]. Initial staging, Breslow index and complete surgery with lymphadenectomy are the main independent variables for survival that have been reported [14,15,16].

It is believed that MMs differ from CMs in molecular profile, as the primary risk factor of CM, sun exposure, does not play a role in the development of mucosal melanomas. BRAF mutations, frequently seen in CM, are not associated with MM. Therefore, there is the necessity to explore molecular pathways altered in MM. In addition, molecular profiling will help in the development of specific treatments for MM. 

Current clinical practice guidelines do not include a specific section for MM. BRAF status is the only validated predictive biomarker at the moment. Most conclusions are based on case reports. Melanoma patients routinely receive either a combination of BRAF/MEK inhibitors or immunotherapy with antiPD1/DPL1 alone or in combination with antiCTLA4; the best sequence is still under discussion. Personalized systemic therapies targeting, for example, KIT mutations are only possible under clinical trials. 

Analyzing results from the literature in the molecular landscape [17,18,19,20], the basic biology of MM still remains unclear, but improvements have been made in recent years. Newell et al. performed the largest study published so far with WGS-analysis of 67 mucosal melanomas from Europe, Australia and China [17]. They confirmed that mucosal melanomas show low contribution from the UVR-signature. Interestingly, patients with somatic mutation in BRCA genes had no germline translation, which has yet to be analyzed in our cohort. They identified a total of 10 significantly mutated genes: NRAS (12/67), BRAF (11/ 67), NF1 (11/67), KIT (10/67), SF3B1 (8/67), TP53 (6/67), SPRED1 (5/67), ATRX (4/67), HLA-A (4/67) and CHD8 (3/67). NRAS mutations were targeted on hotspots of codon 61, which is also seen in our cohort and in what Mikkelsen et al. published afterwards [7].

Alterations in KIT and NF1 are more frequent than in CM, whereas the MAPK-pathway typically including Ras/Raf/MEK/ERK is less dominant. The mutations observed in the BRAF gene in MM affect regions other than codon 600, which are known to lead to weaker MAPK-pathway activation and therefore are not predicted to respond to BRAF inhibition therapies, which are by the way the standard of care in CM [20]. 

KIT is already an established therapeutic target agent in other cancers, specifically in gastrointestinal stromal tumors (GIST). Identification of these known mutations in patients with MM may take into consideration KIT inhibitor treatments [21]. In a trial in which imatinib was used to treat 24 patients with either KIT-mutated or KIT-amplified tumors in mucosal, acral or chronically sun-damaged melanoma, the authors concluded that it was effective in KIT-mutated tumors but not in those where the gene was amplified only [22]. In our study, we found L576P exon 11 on KIT mutation, which is frequent in GIST, suggesting that the molecular profile may indicate target therapies such as imatinib for selected patients. 

NRAS Q61 mutations are typically seen in MM, as we confirmed in our study, and are associated with a poor prognosis and a potential cause of BRAF inhibitor resistance. MEK inhibitors may be effective in these patients. The MEK inhibitor binimetinib has shown activity in this setting, with a response rate of 20% in a Phase II trial of 30 patients with NRAS-mutated melanoma [23]. 

NF1 is a tumor suppression gene. Loss of NF1 is associated with increased MAPK activity and is significantly mutated both in CM and MM. Similarly to NRAS mutations, alterations in NF1 result in poor response to BRAF inhibitors and may be targeted by MEK inhibitors [20].

A limitation of our study is the small size of cases that were suitable for NGS that did not allow solid conclusions to be drawn. We assume that old FFPE tissues harbor high levels of degradation of DNA, and that produces artifacts that complicate the interpretation of NGS results. Part of these artifacts could be eliminated before preparing the libraries, using uracil-DNA glycosylase or nuclease S1 [24,25]. Nevertheless, with this study we have contributed to the understanding of molecular pathways in MM, but more genetic research needs to be done. 

There were six cases for which we could not find any pathogenic mutation. Since MMs carry at least one well-established driver mutation, there is a possibility that we did not detect them because our panel was limited to 44 genes. For example, SF3B1 or SPRED1, described in other articles, were not covered in our panel [7,17,18]. However, there are several copy number variations in mucosal melanoma, not all detected in our study, which can also explain the negative cases, and some detected that do not have a proper interpretation yet [7,17]. 

Today, immune checkpoint inhibitors are the standard of care for many cancer types, including CMs, and have incredibly improved patients’ chance of survival. Three mucosal melanomas respond less to immunotherapy [7,26] than CMs. A postulated reason for this is that the mutation burden is much lower in mucosal melanoma as compared to cutaneous melanoma [20]. Angelo et al. evaluated the efficacy of ipilimumab and nivolumab alone or in combination. The study included data from several clinical studies with 889 melanoma patients, 10% of which had mucosal melanoma; the response rate was 37.5% and the progression-free survival was 5.9 months [27]. In our cohort, two patients with vulvar melanoma received anti-PD1/PDL1 treatment and one patient with nasal melanoma received anti-CTLA4; none of them responded. All other patients who needed systemic treatments received conventional chemotherapy due to the antiquity of our cohort. 

## 5. Conclusions

Population-based data are essential for the understanding of biological behavior in MM and the lack of clinical evidence in treating patients. Treatments for mucosal melanomas are often extrapolated from data based on therapies for metastatic cutaneous melanoma, but knowing and understanding its mutational profile will allow us to design better treatment strategies in the context of more precise medicine. 

Our study supports the steady incidence and poor patient survival of mucosal melanoma in a region of southern Europe. NRAS and NF1 are confirmed to play a role in mucosal melanoma. We believe target therapies may be a very good option to a not-underestimated group of patients with certain actionable mutations.

## Figures and Tables

**Table 1:** Gender distribution and incidence rates of mucosal melanoma in Girona 1994–2018.

Characteristics				CR (per 100.000) (95% CI)			ASIRe (per 100.000) (95% CI)			ASIRw (per 100.000) (95% CI)		
Site	*n* (%)	M/F (%)	Med Age [IQR]	M	F	Total	M	F	Total	M	F	Total
Head & Neck	12 (28.6)	6/6	72.5 [57.0–91.2]	0.07 (0.01–0.13)	0.07 (0.01–0.13)	0.07 (0.03–0.11)	0.09 (0.03–0.22)	0.08 (0.03–0.17)	0.09 (0.05–0.16)	0.05 (0.02–0.15)	0.025 (0.01–0.13)	0.04 (0.02–0.09)
Rectal	15 (35.7)	6/9	69.9 [65.9–82.3]	0.07 (0.01–0.13)	0.11 (0.04–0.18)	0.09 (0.05–0.14)	0.09 (0.03–0.23)	0.10 (0.05–0.20)	0.10 (0.06–0.17)	0.05 (0.02–0.15)	0.036 (0.01–0.14)	0.04 (0.02–0.10)
Vulvar-vaginal	14 (33.3)	0/14	64.4 [57.8–75.4]	--	0.17 (0.08–0.26)	0.09 (0.04–0.13)	--	0.18 (0.10–0.30)	0.09 (0.05–0.16)	--	0.10 (0.05–0.22)	0.05 (0.03–0.11)
Urethral	1 (2.4)	1/0	69.8	0.01 (0–0.04)	--	0.01 (0–0.02)	0.01 (0–0.13)	--	0.01 (0–0.04)	0.008 (0–0.106)	--	0.004 (0–0.054)
All	42 (100)	13/29	68.4 [59.9–84.0]	0.16 (0.07–0.24)	0.36 (0.23–0.48)	0.26 (0.18–0.33)	0.19 (0.10–0.36)	0.36 (0.24–0.52)	0.29 (0.21–0.40)	0.10 (0.05–0.22)	0.16 (0.10–0.29)	0.14 (0.09–0.21)

N: Number of cases; M: males; F: females; CI: confidence interval; CR: crude rate; ASIRe: European age-adjusted standard incidence rate ASIRw; world age-adjusted standard incidence rate and IQR: interquartile range.

**Table 2:** Five-year observed survival for all stages of mucosal melanoma in Girona 1994–2018. OS: observed survival; NA: not applicable.

Site	5y OS (%) (95% CI)		
	Males	Females	Total
Head and Neck	16.7 (2.8–99.7)	33.3 (10.8–100)	25.0 (9.4–66.6)
Rectal	0	33.3 (13.2–84.0)	20.0 (7.3–55.0)
Vulvovaginal	NA	35.7 (17.7–72.1)	NA
All	7.7 (1.2–50.6)	34.5 (20.9–56.9)	26.2 (15.8–43.5)

**Table 3:** Clinical characteristics of patients with vulvar-vaginal mucosal melanoma.

Characteristics	N = 14 (33%)
**Pathology**	
Breslow	
<1 mm	1 (7.1)
1–2 mm	0
2–4 mm	3 (21.4)
>4 mm	10 (71.5)
Ulceration	
Positive	8 (57.2)
Negative	5 (35.7)
Missing	1 (7.1)
**Initial treatment**	
Local surgery only	7 (50)
Local surgery + lymphadenectomy	3 (21.4)
Local surgery + lymphadenectomy + adjuvant radiotherapy	2 (14.3)
Local surgery + lymphadenectomy + adjuvant interferon	1 (7.1)
Radical radiotherapy	0
Systemic treatment only	1 (7.1)
Palliative treatment only	0
Sentinel node biopsy	5 (35.7)
**Stage information (TNM 7th Edition)**	
Stage IB	2 (14.3)
Stage IIA	2 (14.3)
Stage IIB	1 (7.1)
Stage IIC	6 (42.9)
Stage IIIB	2 (14.3)
Stage IIIC	1 (7.1)
**Vital status (at 31 of August 2021)**	
Alive without disease	3 (21.4)
Alive with disease	0
Deceased for specific disease	10 (71.5)
Deceased for all causes	1 (7.1)

**Table 4:** Results from the genetic profiling of the 24 mucosal melanoma samples analyzed.

Mutation/Site	Rectum									Nasal					Pharynx			Vulvovaginal						
	1	4	6	7	9	16	17	21	23	2	3	10	19	20	8	12	18	5	11	13	14	15	22	24
BRAF G596R																								
KRAS G12C																								
NRAS Q61 H/K/R																								
KIT L576P																								
NF1 L1611T																								
NF1 Y1401 *																								
NF1 A1660 *																								
NF1splice acceptor																								
NF1 I766 *																								
NF1splice donor																								
NF1 F624V																								
TP53 S241C																								
TP53 F134L																								
TP 53 R273L																								
TP 53 K139E																								
CDKN2A E10 *																								
MYC F7L																								
BRCA2 E1734K																								
BRCA2 D935N																								
BRCA1 G1777P																								
APC E892G																								
AKT3 A21L *																								
PTEN V166Sfs * 14																								
FGFR3 R158Q																								
MAP2K2 R74S																								
POLE R2165H																								
POLE M1748L																								
POLE R1294C																								
POLD1splice donor																								
ERBB2 V153L																								
ERBB3 R1077W																								
ROS1 M710T																								
MET R359Q																								
JAK2 N1108S																								
STK11 V66G *																								
Wildtype in assay																								

MissenseNonsense→Frameshiftsplice site * Stop codon; Blank cell = no mutation found.

## Data Availability

Data are available at Genetic Descriptive, Genetic and Prevention Epidemiology Group, Biomedical Research Institute of Girona (IDIBGI). This study was carried out using anonymized data from the Girona Cancer Registry (GCR), which complies with the legal regulations in Law for Data Protection and management of clinical data in force in Spain. The GCR also belongs to and complies with the regulations and rules of the International Association of Cancer Registries and the International Association for Research in Cancer (IARC) Cancer Registry (IACR) and the International Association for Cancer.
